# Efficacy of Navigated Laser Photocoagulation for Chronic Central Serous Chorioretinopathy: A Retrospective Observational Study

**DOI:** 10.1155/2022/7792291

**Published:** 2022-04-20

**Authors:** Fen Zhou, Jin Yao, Qin Jiang, Weihua Yang

**Affiliations:** Nanjing Medical University Affiliated Eye Hospital, China

## Abstract

*Background*. No consensus has been reached regarding the management of central serous chorioretinopathy (CSCR). We aimed to investigate the efficacy of navigated laser treatment for chronic CSCR and changes in the morphology of the retinal pigment epithelium (RPE). In this retrospective observational study, 19 patients with subjective symptoms admitted to the Nanjing Medical University Affiliated Eye Hospital were included between January 2021 and August 2021. All patients underwent visual acuity and optical coherence tomography (OCT) examination during follow-up. Fluorescein angiography (FA) was performed at baseline and at the final follow-up to confirm the dye leakage sites. The mean logMAR BCVA at baseline and at the end of follow-up was 0.49 ± 0.24 and 0.24 ± 0.15, respectively. The mean logMAR BCVA 3 months after treatment was significantly better than that before treatment (*p* = 0.002). Significant improvements were observed in central macular thickness (CMT) and subfoveal choroidal thickness (SFCT) after navigated laser photocoagulation (*p* < 0.0001). Subretinal fluid was completely resolved in 13 (68%) of 19 eyes at 3 months. RPE lesions on OCT images showed RPE detachment in 17 eyes (61.5%), small protrusion of the RPE layer in 5 eyes (7.5%), and a rough RPE layer in 4 eyes (31%). After laser treatment, 9 points (35%) showed retinal detachment, and 10 (38.5%) regions showed an irregular RPE layer. An irregularly protruded RPE layer was also observed in different regions of the leakage spot in 5 (19%) eyes, and RPE defects were seen in 2 (7.5%) eyes. Navigated laser photocoagulation for chronic CSCR can achieve substantial anatomical and visual improvement. OCT outcomes may provide new information to facilitate understanding of the mechanism of chronic CSCR. Navigated laser photocoagulation should be chosen as an optimal treatment option in patients with chronic CSCR who cannot afford photodynamic therapy.

## 1. Introduction

Central serous chorioretinopathy (CSCR) is one of the most common retinal diseases characterized by fluid leakage. CSCR is a main cause of vision loss among middle-aged males due to photoreceptor and retinal pigment epithelium (RPE) atrophy [1]. However, the pathogenesis of CSCR remains poorly understood. Fluorescein angiography (FA) shows single or multiple spots of fluorescein leakage at the level of the RPE. Dysfunction in RPE activity and increased vascular choroidal hyperpermeability are considered important factors in fluid collection. RPE dysfunction cannot prevent diffusion of fluid into the subretinal space. Complete resolution of subretinal fluid (SRF) should be the principal target of CSCR therapy, as prolonged SRF can result in permanent damage to the photoreceptors [2]. SRF is a common hallmark of CSCR. The principal monitoring device of SRF is optical coherence tomography (OCT). The volume of SRF can be quantified by OCT. A higher SRF volume may be closely related to poorer best-corrected visual acuity (BCVA) [3]. SRF absorption is an important prognostic indicator in patients with CSCR. If SRF resolves quickly, patients with CSCR have good outcomes. The RPE can be not only impaired by SRF but also damaged by underlying multifocal choroidal vascular dysfunction without the presence of SRF [4, 5]. Changes in central macular thickness (CMT) appear to be associated with a decrease in SRF [6].

To date, there is no consensus regarding the management of CSCR. Treatment options include pharmacology, an intravitreal injection of anti-vascular endothelial growth factor (VEGF) antibodies, and laser photocoagulation. Conventional lasers, subthreshold lasers, photodynamic therapy, and transpupillary thermotherapy are available for laser photocoagulation [7, 8]. CNV secondary to CSCR was treated with the use of anti-VEGF therapy or with the use of PDT [9, 10]. Laser photocoagulation was the first-line treatment in CSCR.

It can be challenging to perform conventional laser photocoagulation for leaks in CSCR. It can be a difficult task to determine the exact location on the live fundus view of the leaky spot among the network of blood vessels on a moving eye. To improve the accuracy and predictability of retinal laser photocoagulation for CSCR, the NAVILAS® laser system (OD-OS GmbH, Teltow, Germany) provides the option to plan the treatment beforehand directly on the early fluorescein angiogram. The angiogram is imported into the laser device, superimposed, and continuously aligned with the live image using an eye tracking system, so that physicians can perform more precise targeted treatments than conventional manually directed laser photocoagulation in CSCR.

The difficulty is alleviated by using a navigated laser device when seeking the leaky spot on the live inverted fundus view through the slit-lamp laser device. Several advantages exist for the NAVILAS® laser system, including identifying the exact location of the leak, eye tracking, and laser planning abilities of the laser system on fluorescein angiograms. In addition, navigated laser photocoagulation is a shorter procedure than conventional laser treatments and is less painful for patients. However, direct observation of precise morphologic changes in the RPE after navigated laser treatment in patients with CSCR has not been conducted. Therefore, this study aimed to evaluate the efficacy of navigated laser treatment for CSCR and to assess morphological changes in the RPE during treatment.

## 2. Materials and Methods

### 2.1. Ethics Statements

This study was approved by the Nanjing Medical University Affiliated Eye Hospital institutional review board and conducted in accordance with the Declaration of Helsinki (1964). Informed consent was obtained from all patients.

### 2.2. Study Design and Patients

In our retrospective observational study, we included 19 eyes of 19 patients with serous retinal detachment who were diagnosed with chronic CSCR. All patients received navigated laser photocoagulation therapy between January 2021 and August 2021 at our hospital. Diagnosis of CSCR was based on fundus examination, FA, and indocyanine green angiography (ICGA). Patients were excluded if diagnosed with intraocular inflammation, retinal vascular occlusive disease (RVO), or neovascular maculopathies such as age-related macular degeneration (AMD) and polypoidal choroidal vasculopathy (PCV). All patients underwent ophthalmic examinations, including slit-lamp microscope examination, measurement of BCVA, Goldmann tonometry, spectral-domain OCT (Spectralis HRA+OCT, Heidelberg Spectralis, Heidelberg Engineering GmbH, Heidelberg, Germany) of the macula, and FA (Spectralis HRA+OCT, Heidelberg Spectralis, Heidelberg Engineering GmbH).

### 2.3. Treatment

Before laser photocoagulation using the NAVILAS® laser system (532 nm double-pulsed YAG laser, OD-OS GmbH), an image of the posterior pole was captured. The physician planned treatment by using the early images of FA that showed a leaking spot, and then, the same physician performed the laser treatment. To determine the parameters for the laser shots, an initial test burn away from the macular region was performed. Laser parameters were set as follows: grayish white burn, energy 50-80 mW; spot size, 100-150 *μ*m; and pulse duration, 70-100 ms. One to ten laser spots of 100-150 *μ*m per leaking point were marked on the superimposed image together with a protection shield for the fovea and optic nerve. After laser treatment, all patients underwent color fundus photography. The laser software provided an additional feedback window that showed the immediate laser effect captured at the end of the pulse. Treatment was repeated if focal leakage of the same lesion was visible on FA at the 1-month follow-up. If patients developed a new leaking point on FA distant from the previous lesion, they also received additional laser treatment. This was considered as a second treatment of the same eye.

### 2.4. Follow-Up and Analysis

Comprehensive eye examinations were performed during every follow-up visit, such as OCT and FA. All patients were examined at 2 weeks, 4 weeks, and 3 months after laser treatment. Data regarding BCVA, subfoveal choroidal thickness (SFCT), and CMT were collected from the records of the patients in this study. At baseline, CMT was measured from the inner surface of the neurosensory retina to the inner portion of the RPE at the fovea. SFCT was measured via subfoveal localization using enhanced depth imaging [11].

### 2.5. Statistical Analysis

Each variable involved calculation of descriptive statistics. Data were expressed as the mean ± SD. Normal distribution was checked by using the Shapiro-Wilk test. Paired *t*-test were used to analyze the changes in BCVA at baseline and follow-up at 2 weeks, 4 weeks, and 3 months. CMT and SFCT differences between baseline and 3-month follow-up were also calculated by paired *t*-test. Statistical analysis was performed using SPSS Statistics version 22 (IBM, Armonk, NY). Statistical significance was set at *p* < 0.05.

## 3. Results

### 3.1. Patients

Table 1 shows the patient characteristics. In total, 19 eyes of 19 patients (15 men (78.9%) and 4 women (21.1%); mean age, 41.7 ± 8.6 years; age range, 31–65 years) were enrolled in this study. The mean duration of symptoms was 13 ± 15 months (range, 3 months to 4 years). Thirteen eyes (74%) had one point of focal leakage, four eyes (21%) showed two points of leakage, and one eye (5%) showed four points of leakage. Dye leakage was observed in all patients on FA. Seventeen and two patients presented with inkblot and smokestack leakages on FA, respectively. Two eyes had previously received treatment with a subthreshold micropulse laser (SML), and four eyes had a history of CSCR in the fellow eye.

### 3.2. Visual Acuity

**Figure 1:**
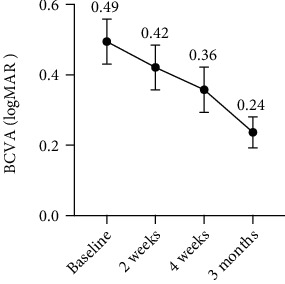
Variation of best-corrected visual acuity (logMAR) during follow-up expressed as mean and standard deviation. The comparison between different time points was evaluated. We found a time-dependent increase in BCVA. The mean logMAR BCVA was 0.42 ± 0.21 and 0.36 ± 0.22 at 2-week and at 4-week follow-up, respectively. There was no statistically significant difference compared to baseline (*p* = 0.4192, *p* = 0.1403). There was a sharp increase at 3-month follow-up. LogMAR BCVA at 3-month follow-up was higher than at baseline. The difference was significant (*p* = 0.002).

The mean logMAR BCVA at baseline was 0.49 ± 0.24. At the final visit, the mean logMAR BCVA was 0.24 ± 0.15. The mean logMAR BCVA at 2 weeks and 4 weeks was 0.42 ± 0.21 and 0.36 ± 0.22, respectively (Figure 1).

### 3.3. Findings of Optical Coherence Tomography

**Figure 2:**
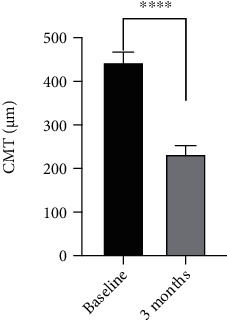
The mean CMT value at the 3-month follow-up was higher than at baseline. The deference was significant. ^∗∗∗∗^*p* < 0.0001.

**Figure 3:**
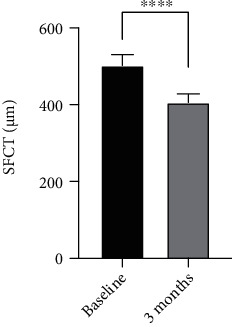
The mean SFCT value at the 3-month follow-up was higher than at baseline. The deference was significant. ^∗∗∗∗^*p* < 0.0001.

**Figure 4:**
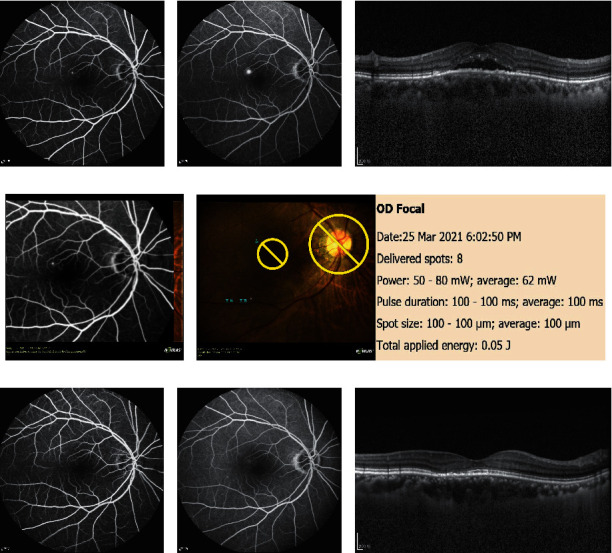
Images of a 65-year-old woman with chronic CSC for 48 months. (a) FA showed early hyperfluorescence representing a leak. (b) The hyperfluorescence was increased in the late phase. (c) OCT showed obvious subretinal fluid and irregular RPE. (d) The selected FA image showing the focal leakage was imported and automatically superimposed on the color fundus photograph. (e) NAVILAS® laser photocoagulation was programmed on the actual image to three single laser spots on the leak and performed with the following parameters: spot size: 100 *μ*m; power: 50–80 mW; and pulse duration: 100 ms. (f) At the 3-month follow-up, focal leakage in the early stage on FA has disappeared. (g) The focal leakage in the late stage on FA has also disappeared. (h) OCT showed no serous pigment epithelial detachment.

Figures 2 and 3 show the outcome parameters. At baseline and at the 3-month follow-up, the mean CMT values were 443.9 ± 79.5 *μ*m and 232.9 ± 48.4 *μ*m, respectively, while the mean SFCT values were 502.6 ± 96.6 *μ*m and 406.3 ± 79.7 *μ*m, respectively. Figures 4(a)–4(h) show a representative case.

### 3.4. Changes in Subretinal Fluid during the Follow-Up

**Figure 5:**
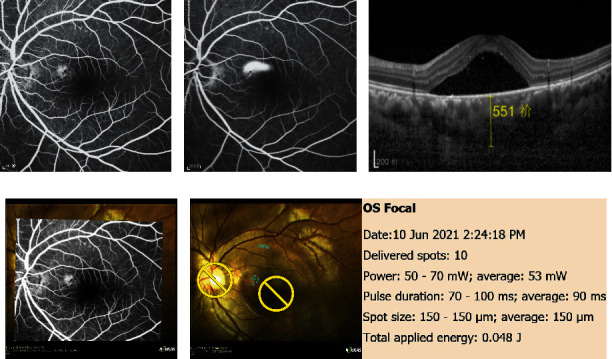
Images of a 40-year-old male patient with recurrent and chronic central serous chorioretinopathy for 4 months. (a) FA showed focal leakage in the early stage. (b) FA showed more obvious focal leakage in the late stage. (c) OCT showed conspicuous serous pigment epithelium detachment and the thickness of choroid was 551 *μ*m. (d) The selected FA image showing the focal leakage was imported and automatically superimposed on the color fundus photograph. (e) NAVILAS® laser photocoagulation was programmed on the actual image to seven single laser spots on the leak and performed with the following parameters: spot size: 150 *μ*m; power: 50–70 mW; and pulse duration: 70–100 ms.

SRF was completely resolved in 13 (68%) of 19 eyes at 3 months. However, 6 leaky points (23%) among 26 leakage sites were also detected in 6 eyes. Extensive SRF was present and involved the macula. Figures 5(a)–5(d) show a representative case.

### 3.5. Changes in the Retinal Pigment Epithelium during the Follow-Up

**Figure 6:**
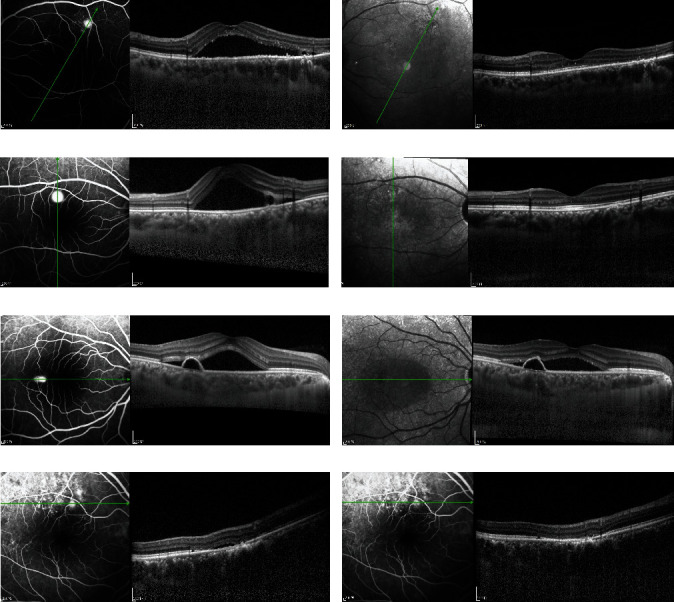
(a) OCT image showed SRF and irregular RPE. (b) OCT image shows an RPE defect and complete absorption of SRF after 3 months. (c) OCT image showed the retina dipping above the irregular RPE with slight fibrin. (d) After 3 months of NAVILAS® laser photocoagulation, OCT image shows an RPE defect and complete absorption of SRF. (e) OCT image showed SRF and PED at the dye leakage spot. (f) OCT image after 3 months of NAVILAS® laser photocoagulation showed decreased SRF, but PED remains. (g) OCT image showed irregular RPE changes. (h) After 3 months of NAVILAS® laser photocoagulation, OCT image also showed irregular RPE changes but complete absorption of SRF.

Among the 26 leakage sites in 19 eyes, 16 (61.5%) showed RPE detachment (PED), and 8 (31%) showed an irregular RPE layer. An irregularly protruded RPE layer was also observed in different regions from the leakage spot in two eyes (7.5%). After laser treatment, 9 points (35%) showed PED, and 10 (38.5%) regions showed an irregular RPE layer. The irregularly protruded RPE layer was also observed in different regions from the leakage spot in five (19%) eyes, and RPE defects were observed in two (7.5%) eyes. Figures 6(a)–6(h) show a representative case.

## 4. Discussion

Although there are various available treatment options for CSCR, laser-related RPE atrophy and choroidal neovascularization cannot be ignored [12, 13]. Navigated laser photocoagulation may be an optimal option, especially during the coronavirus disease pandemic. A previous report showed that navigated laser photocoagulation was safe and more accurate (92%) than a conventional laser (72%) in the treatment of diabetic retinopathy microaneurysms [14]. Recently, resolution of SRF and minimum iatrogenic damage was achieved through navigated laser photocoagulation in CSCR [15]. Therefore, it was deemed an effective and safe alternative therapy for patients with chronic CSCR [16].

Half-dose photodynamic therapy is superior to SML for treating CSCR regarding the complete resolution of SRF and functional improvement. At 6–8 weeks after treatment, resolution of SRF was achieved in 51.2% and 13.8% of patients, respectively [17]. In our study, the outcomes were also favorable. The mechanism of SRF resolution after laser photocoagulation treatment is yet unknown. In previous studies, some mechanisms like stimulating the pump function of RPE cells near the leak, sealing focal defects in the RPE, promoting a healing response, and recruiting healthy RPE cells have been proposed [18–21]. In CSCR, the prolonged presence of SRF can cause decreased vision and irreversible RPE damage. Our study showed an improvement in BCVA from 0.37 to 0.62 after NAVILAS® laser photocoagulation treatment by follow-up at 3 months. Similar BCVA outcomes were reported by Müller et al. in 32 previously untreated eyes after 3 months [16]. SRF can be used as an important biomarker of CSCR, and we found that navigated laser photocoagulation could induce complete resolution of SRF in 68% of patients with CSCR in the short term.

The pathological mechanism of CSCR includes enhanced permeability of choroidal capillaries and increased hydrostatic pressure, which leads to abnormal morphology and decreased function of the RPE and damage to its barrier function, leading to the infiltration of plasma and other substances in choroidal capillaries into the retinal neuroepithelium [1, 22, 23]. The mechanism of CSCR can be better understood with OCT, especially the changes in the RPE layer [24, 25].

To the best of our knowledge, this is the first report to draw attention to RPE morphological changes after navigated laser photocoagulation treatment in CSCR. Our study showed OCT images of leaky spots at the level of the RPE. Before laser photocoagulation treatment, RPE changes were found in the leaky regions of affected eyes with PED, an irregular RPE, a protruding RPE, and RPE defects. OCT images of leakage points showed PED in 16 leaky points (61.5%), small protrusion of the RPE layer at 2 leaky points (7.5%), and a rough RPE layer at 8 leaky points (31%). Mitarai et al. reported similar RPE changes [26]. PED may be the most common RPE abnormality at the leaky point in eyes with CSCR. Hirami et al. reported that RPE abnormalities within areas of choroidal vascular hyperpermeability were observed [27]. Dysfunction of the underlying choroid is likely to impair barrier function. Fluid can pass from the sub-RPE to the subretinal area due to a defect in the RPE layer. Thermal photocoagulation of the leaking site or region of RPE dysfunction may seal the leaky points and stimulate the neighboring RPE cells covering the coagulated area. Our study showed OCT changes in the RPE after thermal photocoagulation. PED was significantly reduced, and RPE morphology changed to an irregular RPE and a protruded RPE. This suggests that laser photocoagulation therapy is helpful for the gradual recovery of the morphology and function of the RPE. However, nine points (35%) showed retinal PED in seven eyes. Repeated laser treatment was performed in six patients with six leaky points, although RPE morphological change consisted of PED in five leaky points. This emphasized the importance of maintaining RPE integrity against increasing pressure from the choroid capillaries.

Eight regions (31%) showed an irregular RPE layer before the laser treatment. When a defect occurs in abnormal RPE regions, a new leakage spot will be observed. After laser treatment, an irregular RPE will change to an irregular and protruded RPE with RPE defects. In our study, an irregularly protruded RPE layer was also observed in different regions from the leakage site in two eyes (7.5%). The protruded RPE changed to an irregular and protruded RPE. This result shows that laser photocoagulation could promote the absorption of SRF; however, localized RPE defects and irregular RPE changed to an abnormal RPE morphology, indicating repair of functional damage of the RPE. Therefore, maintaining the integrity of RPE morphology is very important to resist the increased hydrostatic pressure of choroid capillaries during CSCR treatment.

Choroidal hyperpermeability at the location of the RPE leakage can be observed in ICGA. An enhanced-depth imaging model (EDI) of the choroid is another aspect of this study. Studies have revealed that there will be a reduction in SFCT with the resolution of the disease [28, 29]. Our study showed that the median value of SFCT was 502.6 mm (range, 326-757 mm) at baseline. After navigated laser photocoagulation, the SFCT decreased to 406.3 mm. This suggests that choroidal thickness may play an important role in the pathophysiology of CSCR.

The limitations of our study are its retrospective design, small sample size, and no control group. To study the efficacy of navigated laser photocoagulation in CSCR would require further studies with a longer follow-up.

## 5. Conclusions

In conclusion, after laser treatment, SRF absorption takes time. This finding draws attention to the development of best practice guidelines for treating CSCR. Navigated laser photocoagulation is a reliable treatment choice for sealing exudative lesions and allowing the resolution of SRF in some patients with chronic CSCR. OCT also provides a better understanding of RPE changes in this disease.

## Figures and Tables

**Table 1:** Patient characteristics.

Case	Age	Gender	Eye	Leakage pattern	Leak point	OCT-ophthalmoscope	VA	CMT (*μ*m)	SFCT (*μ*m)
1	50	M	L	Inkblot	1	Protrusion-protrusion	0.15	663	700
2	52	F	R	Smokestack	1	Rough RPE-protrusion	0.3	466	632
3	54	M	R	Smokestack	2	Rough RPE-protrusion	0.12	374	550
						PED-rough RPE			
4	33	M	L	Inkblot	1	PED-protrusion	0.15	570	546
5	39	M	L	Inkblot	1	PED-PED	0.6	389	398
6	37	M	L	Inkblot	1	Protrusion-rough RPE	0.4	455	455
7	31	F	R	Inkblot	2	PED-PED	0.5	598	757
						PED-rough RPE			
8	38	M	R	Inkblot	1	Rough RPE-RPE defect	0.1	361	326
9	65	F	R	Inkblot	1	Rough RPE-rough RPE	0.4	372	377
10	50	M	L	Inkblot	1	PED-rough RPE	0.3	422	487
11	43	M	R	Inkblot	1	PED-PED	0.2	399	565
12	40	F	R	Inkblot	1	PED-protrusion	0.5	287	465
13	63	M	R	Inkblot	1	PED-PED	0.2	302	482
14	49	M	R	Inkblot	2	PED-PED	0.6	546	554
						PED-PED			
15	38	M	L	Inkblot	2	PED-PED	0.8	374	500
						PED-PED			
16	43	M	R	Inkblot	4	Rough RPE-rough RPE (3)	0.6	410	634
						PED-rough RPE (1)			
17	57	M	R	Inkblot	1	PED-rough RPE	0.4	438	439
18	64	M	L	Inkblot	1	Rough RPE-RPE defect	0.15	500	354
19	49	M	R	Inkblot	1	PED-PED	0.6	508	328

## Data Availability

The article can be obtained from the corresponding author six months after publication.
